# The Involvement of Pial Microvessels in Leukocyte Invasion after Mild Traumatic Brain Injury

**DOI:** 10.1371/journal.pone.0167677

**Published:** 2016-12-28

**Authors:** Joanna Szmydynger-Chodobska, Rongzi Shan, Nicole Thomasian, Adam Chodobski

**Affiliations:** Neurotrauma and Brain Barriers Research Laboratory, Department of Emergency Medicine, Alpert Medical School of Brown University, Providence, Rhode Island, United States of America; University of Florida, UNITED STATES

## Abstract

The pathophysiological mechanisms underlying mild traumatic brain injury (mTBI) are not well understood, but likely involve neuroinflammation. Here the controlled cortical impact model of mTBI in rats was used to test this hypothesis. Mild TBI caused a rapid (within 6 h post-mTBI) upregulation of synthesis of TNF-α and IL-1β in the cerebral cortex and hippocampus, followed by an increase in production of neutrophil (CXCL1–3) and monocyte (CCL2) chemoattractants. While astrocytes were not a significant source of CXC chemokines, they highly expressed CCL2. An increase in production of CXC chemokines coincided with the influx of neutrophils into the injured brain. At 6 h post-mTBI, we observed a robust influx of CCL2-expressing neutrophils across pial microvessels into the subarachnoid space (SAS) near the injury site. Mild TBI was not accompanied by any significant influx of neutrophils into the brain parenchyma until 24 h after injury. This was associated with an early induction of expression of intercellular adhesion molecule 1 on the endothelium of the ipsilateral pial, but not intraparenchymal, microvessels. At 6 h post-mTBI, we also observed a robust influx of neutrophils into the ipsilateral cistern of velum interpositum (CVI), a slit-shaped cerebrospinal fluid space located above the 3rd ventricle with highly vascularized pia mater. From SAS and CVI, neutrophils appeared to move along the perivascular spaces to enter the brain parenchyma. The monocyte influx was not observed until 24 h post-mTBI, and these inflammatory cells predominantly entered the ipsilateral SAS and CVI, with a limited invasion of brain parenchyma. These observations indicate that the endothelium of pial microvessels responds to injury differently than that of intraparenchymal microvessels, which may be associated with the lack of astrocytic ensheathment of cerebrovascular endothelium in pial microvessels. These findings also suggest that neuroinflammation represents the potential therapeutic target in mTBI.

## Introduction

Traumatic brain injury (TBI) is a global public health problem [1–3]. Estimated 70–90% of TBI cases represent mild TBI (mTBI), which is often considered synonymous with concussion. While both adults and children who have sustained a concussion generally recover within three months after injury, there is a substantial subset of individuals having delayed recovery [4–7], which results in loss of productivity, learning difficulties, and psychosocial distress. It is quite likely that these individuals would benefit from appropriate medical intervention, but no concussion-specific treatment is currently available.

The pathophysiological processes associated with mTBI that could be targeted therapeutically are not well defined. Recently identified blood biomarkers for the diagnosis of concussion [8], such as matrix metalloproteinase 9 (MMP9) and galectin 3, whose synthesis is upregulated in response to injury, may represent potential targets for therapeutic intervention in mTBI. Studies of rodent models of focal and diffuse mTBI [9,10] suggest that mTBI is accompanied by neuroinflammation. There is substantial evidence based on data obtained from animal models of severe TBI that therapies directed against neuroinflammation, in particular those limiting the influx of inflammatory cells, reduce the post-traumatic loss of neural tissue and improve functional outcome after injury [11–15]. In the above-mentioned rodent studies of mTBI, changes in production of proinflammatory mediators caused by injury were analyzed, but these investigations did not provide information on possible leukocyte trafficking into the injured brain. The post-injury influx of inflammatory cells was anticipated based on increased synthesis of neutrophil chemoattractants (CXCL1 and CCL3) found in the models of both focal and diffuse mTBI [10]. This question was addressed in the present study in which a rat model of focal mTBI was employed that was similar to the model used by Redell et al. [10].

## Materials and Methods

### Rats

Adult male Long-Evans rats weighing 250–300 g (Harlan, Indianapolis, IN) were used. The rats were kept at 22°C with a 12-h light cycle and maintained on standard pelleted rat chow and water *ad libitum*.

### Reagents and antibodies

ThermoScript RNase H^−^reverse transcriptase and RNase inhibitor RNaseOut were obtained from Invitrogen (Carlsbad, CA). HotStart *Taq* DNA polymerase was purchased from Qiagen (Valencia, CA). The following rabbit polyclonal antibodies were used: anti-rat CCL2 (1 μg/mL) from Antigenix America (Huntington Station, NY); anti-human myeloperoxidase (MPO; 13.2 μg/mL) and von Willebrand factor (vWF; 10 μg/ml) from Dako (Glostrup, Denmark). The following mouse monoclonal antibodies were used: anti-rat CD68 (clone ED1; 1 μg/mL), CD11b (clone MRC OX-42; 1 μg/mL), and RECA-1 (clone HIS52; 5 μg/mL) from Serotec (Oxford, UK); anti-porcine glial fibrillary acidic protein (GFAP; clone GA5; 0.1 μg/mL) from Chemicon International (Temecula, CA); anti-bovine S100B (clone 4C4.9; diluted 1:200) and anti-human intercellular adhesion molecule 1 (ICAM1; clone MEM-111; 10 μg/mL) from Abcam (Cambridge, MA); anti-rat P-selectin (SELP; clone RP-2; 50 μg/mL) [16], a generous gift from Dr. Andrew Issekutz (Department of Pediatrics, Dalhousie University, Halifax, Nova Scotia, Canada). Secondary antibodies were obtained from Molecular Probes (Eugene, OR). These were goat anti-rabbit and anti-mouse antibodies conjugated with Alexa Fluor 488 or 594. They were used at a concentration of 2 μg/mL.

### The rat model of mTBI

The surgical and animal care procedures used in this study were approved by the Animal Care and Use Committee of Rhode Island Hospital and conformed to international guidelines on the ethical use of animals. The controlled cortical impact (CCI) model of mTBI was employed. The rats were anesthetized with intraperitoneal pentobarbital sodium (60 mg/kg) and a 4-mm craniotomy was performed on the right side of the skull to expose the dura, with the center of the opening located 3 mm posterior to the bregma and 2.5 mm lateral to the midline. The velocity of impact was 5 m/sec and the duration of impact was 50 msec. The diameter of the impactor tip was 2.5 mm and the depth of brain deformation was set at 0.5, 1.0, or 1.5 mm. Sham-injured animals underwent all the surgical procedures, as described above, except for injury. At 6 and 24 h after injury, the rats were perfused transcardially with ice-cold 0.9% NaCl and the samples of the cerebral cortex and hippocampus were collected for real-time RT-PCR (*n* = 6 rats per group) and ELISA analyses (*n* = 5–8 rats per group). The animals were under full anesthesia prior to this transcardial perfusion. For immunohistochemistry, the animals (*n* = 4 rats per group) were perfused transcardially with ice-cold 0.9% NaCl, followed by ice-cold 4% paraformaldehyde in 50 mM phosphate-buffered saline, pH 7.4. The brains were collected and post-fixed in the paraformaldehyde solution for 48 h at 4°C.

### Real-time RT-PCR

Total RNA was isolated from cortical and hippocampal samples using NucleoSpin RNA II kit (Macherey-Nagel, Düren, Germany). First-strand cDNAs were synthesized using oligo(dT)_20_ primer (50 pmol) and 15 U of ThermoScript RNase H^−^reverse transcriptase. The 20-μL reverse transcription reactions also contained 40 U of RNase inhibitor RNaseOut. For each reaction, 1 μg of total RNA was used and the reactions were carried out for 1 h at 50°C. Real-time PCR was performed using TaqMan chemistry and the samples were analyzed in duplicate. The sequences of primers and TaqMan probes are provided in Table 1. Peptidylprolyl isomerase A (PPIA)/cyclophilin A was used for the normalization of the data obtained [17]. The 25-μL PCR reaction mixtures contained 0.2 mM mixed dNTPs, 0.2 μM each primer, 0.1 μM TaqMan probe, 5 mM MgCl_2_, 1 U of HotStart *Taq* DNA polymerase, and 1/20 of the reverse transcription reaction product. For PPIA, 1/1000 of the reverse transcription reaction product was used. The reaction mixtures were heated to 95°C for 15 min and were then subjected to 45 cycles of denaturation (15 sec) at 94°C and annealing/extension (60°C, 60 sec).

**Table 1:** The sequences of primers and TaqMan probes, and the predicted sizes of PCR products.

Gene	Primer/probe	Sequence	Predicted size of PCR product (base pairs)
TNF-α	F	5**’**-ATTTCCAACAACTACGATGCTC-3**’**	112
	R	5**’**-GAGTTCCGAAAGCCCATTG-3**’**	
	P	5**’**-CTGGATTGCGGGCTGCTCAT-3**’**	
IL-1β	F	5**’**-CTCAATGGACAGAACATAAGCC-3**’**	143
	R	5**’**-GGTGTGCCGTCTTTCATCA-3**’**	
	P	5**’**-AGAGACAAGCAACGACAAAATCCCTG-3**’**	
CXCL1	F	5’-TGTCCAAAAGATGCTAAAGGG-3’	89
	R	5’-AGAAGCCAGCGTTCACCA-3’	
	P	5’-AAGATAGATTGCACCGATGGCGTC-3’	
CXCL2	F	5**’**-CTGAACAAAGGCAAGGCTAAC-3**’**	120
	R	5**’**-CATCAGGTACGATCCAGGCT-3**’**	
	P	5**’**-CCTGGAAAGGAAGAACATGGGCTC-3**’**	
CXCL3	F	5**’**-CCAGAAGTTACTGAAGAGTGACAAG-3**’**	131
	R	5**’**-CAGGCAGAGGCTCATCCA-3**’**	
	P	5**’**-TGTACGGTGATGGAGGACCTGCC-3**’**	
CCL2	F	5’-TGTCTCAGCCAGATGCAGTTA-3’	181
	R	5’-CATTCCTTATTGGGGTCAGC-3’	
	P	5’-ATGCCCCACTCACCTGCTGCTA-3’	
LCN2	F	5′-CTGTACGGAAGAACCAAGGG-3′	116
	R	5′-CATTGGTCGGTGGGAACA-3′	
	P	5′-CTGTCCGATGAACTGAAGGAGCGAT-3′	
CHI3L1	F	5′-GGTGAAGTACCTGAAGAACAAGC-3′	113
	R	5′-CGTTGGTGAGCGGGAAG-3′	
	P	5′-TGGTGTGGGCAGTGGATTTGGA-3′	
MMP9	F	5’-CTTGAAGTCTCAGAAGGTGGAT-3’	200
	R	5’-GCAGGAGGTCATAGGTCACG-3’	
	P	5’-AGTTCTCTGGCGTGCCCTGGA-3’	
PPIA	F	5**’**-GGTGAAAGAAGGCATGAGCA-3’	152
	R	5**’**-GCTACAGAAGGAATGGTTTGATG-3**’**	
	P	5**’**-TTTGGGTCCAGGAATGGCAAGAC-3**’**	

F, forward primer; R, reverse primer; P, probe.

### ELISA

Proteins from cortical and hippocampal samples were extracted using isotonic lysis buffer (150 mM NaCl, 50 mM Tris-HCl, pH 7.4, 2 mM EDTA, 1% Triton X-100) containing protease inhibitors (1 mM benzamidine, 100 U/mL aprotinin, 20 μg/mL antipain, 20 μg/mL leupeptin, 1 μg/mL pepstatin A, 1 mM PMSF). The samples were analyzed in duplicate. The concentrations of proinflammatory cytokines tumor necrosis factor-α (TNF-α) and interleukin-1β (IL-1β), as well as neutrophil chemoattractants CXCL1–3 were determined using the ELISA DouSet kits from R&D Systems (Minneapolis, MN). The concentration of monocyte chemoattractant CCL2 was measured using the ELISA kit from PeproTech (Rocky Hill, NJ).

### Immunohistochemistry

The coronal brain sections cut on a vibratome at 100 μm were used. Immunohistochemical procedures were performed at room temperature except for the incubation with primary antibodies that was completed at 4°C. To minimize non-specific staining, the brain sections were incubated for 30 min with 10% normal goat serum (Jackson Immunoresearch Labs, West Grove, PA). Four percent of normal goat serum was also added when the sections were incubated with primary and secondary antibodies. After the initial blocking step, the sections were incubated overnight with primary antibodies and then were incubated for 1 h with secondary antibodies. The sections were mounted with Vectashield mounting medium (Vector Labs, Burlingame, CA).

### Statistical analysis

For statistical evaluation of data, ANOVA was used, followed by the Newman-Keuls test for multiple comparisons among means. The results are presented as mean values ± SEM. *p*<0.05 was considered statistically significant.

## Results

### Mild TBI is associated with increased production of proinflammatory mediators

**Fig 1:**
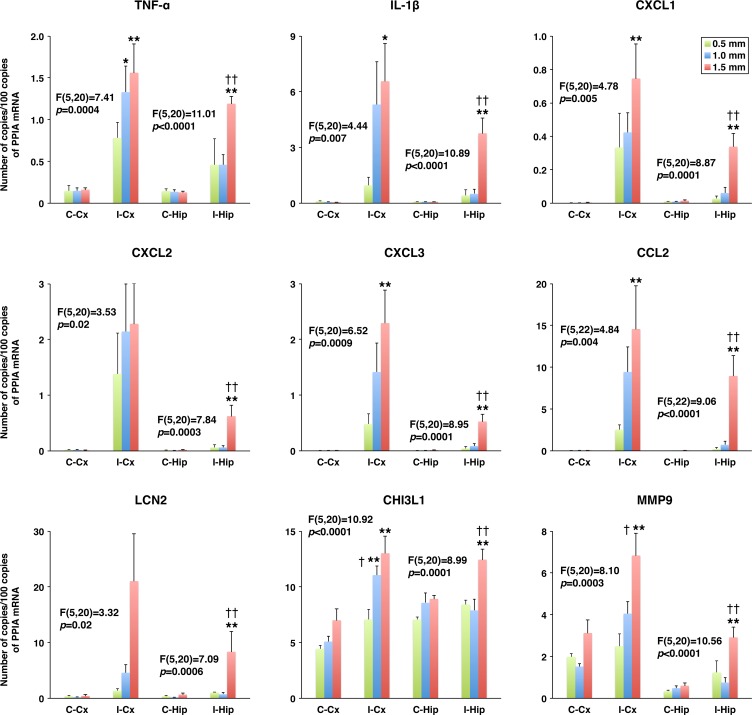
Changes in mRNA levels of proinflammatory mediators in the cerebral cortex and hippocampus at 6 h after mTBI as assessed by real-time RT-PCR. The severity of injury was gradually increased by increasing the depth of brain deformation in 0.5-mm increments from 0.5 to 1.5 mm. The production of proinflammatory mediators in the ipsilateral (I-Cx) versus contralateral (C-Cx) cortex, and the ipsilateral (I-Hip) versus contralateral (C-Hip) hippocampus is shown. Peptidylprolyl isomerase A (PPIA)/cyclophilin A was used for the normalization of the data. The F and the corresponding *p* values for each proinflammatory mediator are indicated. *p<0.05, **p<0.01 for the ipsilateral versus contralateral Cx or Hip. ^†^*p*<0.05, ^††^*p*<0.01 for the difference in expression of proinflammatory mediators in response to a 0.5-mm increase in brain deformation. (*n* = 4–6 rats per group)

**Fig 2:**
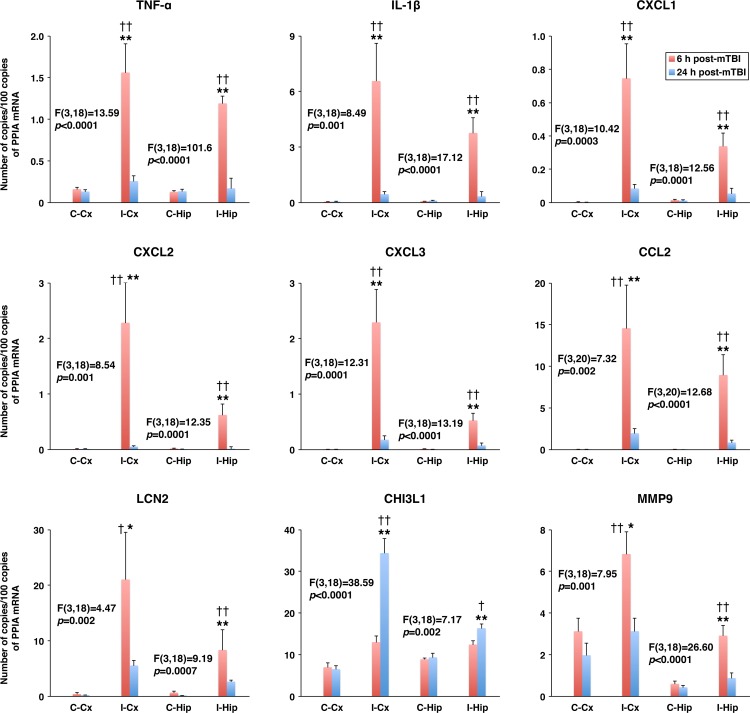
Changes in mRNA levels of proinflammatory mediators in the cerebral cortex and hippocampus at 6 and 24 h after mTBI as assessed by real-time RT-PCR. In these experiments, the depth of brain deformation was set at 1.5 mm. The production of proinflammatory mediators in the ipsilateral (I-Cx) versus contralateral (C-Cx) cortex, and the ipsilateral (I-Hip) versus contralateral (C-Hip) hippocampus is shown. Peptidylprolyl isomerase A (PPIA)/cyclophilin A was used for the normalization of the data. The F and the corresponding *p* values for each proinflammatory mediator are indicated. *p<0.05, **p<0.01 for the ipsilateral versus contralateral Cx or Hip. ^†^*p*<0.05, ^††^*p*<0.01 for the difference in expression of proinflammatory mediators at 6 versus 24 h post-mTBI. (*n* = 4–6 rats per group)

**Fig 3:**
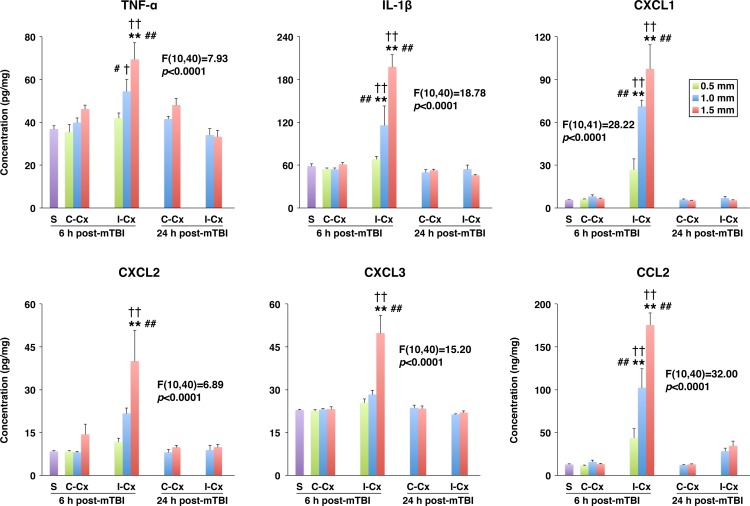
Changes in protein levels of proinflammatory mediators in the cerebral cortex at 6 and 24 h after mTBI as assessed by ELISA. The severity of injury was gradually increased by increasing the depth of brain deformation in 0.5-mm increments from 0.5 to 1.5 mm. The production of proinflammatory mediators in the ipsilateral (I-Cx) and contralateral (C-Cx) cortices, as well as the cortex from sham-injured rats (S), is shown. The F and the corresponding *p* values for each proinflammatory mediator are indicated. **p<0.01 for the ipsilateral versus contralateral Cx. ^#^*p*<0.05, ^##^*p*<0.01 for the difference in expression of proinflammatory mediators from sham-injured rats. ^†^*p*<0.05, ^††^*p*<0.01 for the difference in expression of proinflammatory mediators at 6 versus 24 h post-mTBI. (*n* = 3–8 rats per group)

**Fig 4:**
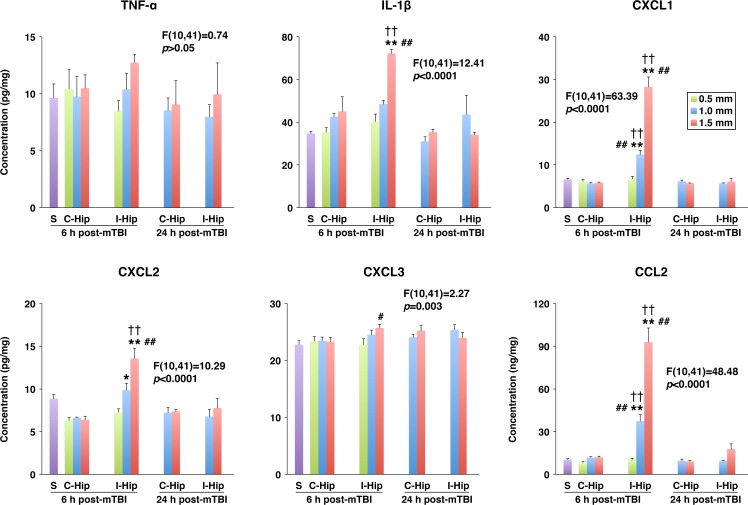
Changes in protein levels of proinflammatory mediators in the hippocampus at 6 and 24 h after mTBI as assessed by ELISA. The severity of injury was gradually increased by increasing the depth of brain deformation in 0.5-mm increments from 0.5 to 1.5 mm. The production of proinflammatory mediators in the ipsilateral (I-Hip) and contralateral (C-Hip) hippocampi, as well as the hippocampus from sham-injured rats (S), is shown. *p<0.05, **p<0.01 for the ipsilateral versus contralateral Hip. The F and the corresponding *p* values for each proinflammatory mediator are indicated. ^#^*p*<0.05, ^##^*p*<0.01 for the difference in expression of proinflammatory mediators from sham-injured rats. ^††^*p*<0.01 for the difference in expression of proinflammatory mediators at 6 versus 24 h post-mTBI. (*n* = 3–8 rats per group)

**Fig 5:**
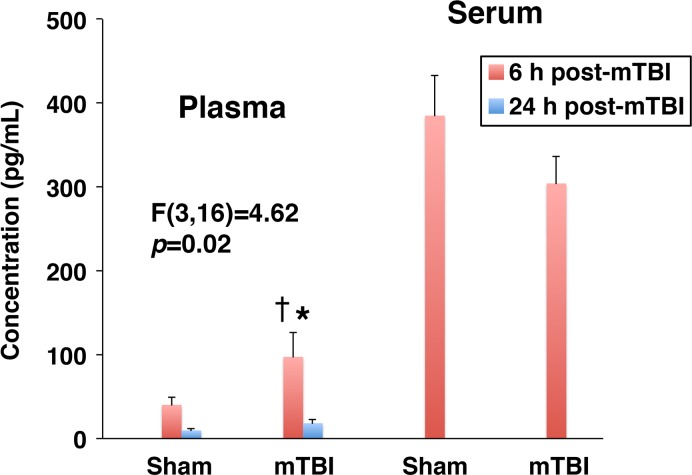
Changes in concentration of neutrophil chemoattractant CXCL1 in peripheral blood plasma and serum at 6 and 24 h after mTBI as assessed by ELISA. In these experiments, the depth of brain deformation was set at 1 mm. Note that sham-injured rats had a high serum level of CXCL1 (~10-fold higher than that observed in plasma), which did not change in response to injury. Other neutrophil chemoattractants investigated (CXCL2, CXCL3, and CCL2) could not be detected in peripheral blood plasma with the immunoassays used in this study. The F and the corresponding *p* values are indicated. *p<0.05 for the difference from sham-injured rats. ^†^*p*<0.05 for the difference in CXCL1 levels at 6 versus 24 h post-mTBI. (*n* = 3–9 rats per group)

In the present study, a rodent model of mTBI, which produces unilateral injury to the cerebral cortex and, to a lesser extent, subcortical structures, was used. In these experiments, the severity of injury was gradually increased by increasing the depth of brain deformation in 0.5-mm increments from 0.5 to 1.5 mm. The real-time RT-PCR analysis (original data are provided in S1 Table) demonstrated a rapid (within 6 h post-mTBI) increase in cortical synthesis (ipsilateral versus contralateral cortex) of proinflammatory cytokines TNF-α and IL-1β (Fig 1). Since TNF-α and IL-1β are well-known inducers of chemokine synthesis, we also examined changes in expression of neutrophil (CXCL1–3) and monocyte (CCL2) chemoattractants after mTBI. Similar to TNF-α and IL-1β, a rapid increase in cortical synthesis of these chemokines was observed, with the magnitude of changes in mRNA expression being dependent on the severity of injury (Fig 1). The cortical expression of other proinflammatory mediators, such as lipocalin 2 (LCN2) and chitinase 3 like 1 (CHI3L1), was also rapidly increased after mTBI (Fig 1). In addition, we found a significant increase in cortical expression of MMP9 at 6 h post-mTBI (Fig 1). The magnitude of changes in expression of these proinflammatory mediators in the ipsilateral hippocampus was smaller than that found in the ipsilateral cortex, except for TNF-α and CHI3L1 for which the post-mTBI increases in mRNA expression in these two brain regions were comparable at the highest severity of injury investigated (Fig 1). Mild TBI was associated with a rapid, but short-lasting, increase in brain synthesis of proinflammatory mediators, and by 24 h post-mTBI, their expression levels in the ipsilateral cortex and hippocampus were similar to those found in the contralateral tissues (Fig 2). The only exception among the proinflammatory mediators investigated in this study was CHI3L1 whose expression levels in both the ipsilateral cortex and hippocampus at 24 h post-mTBI were significantly higher than those observed at 6 h after injury. Changes in cortical and hippocampal expression of these proinflammatory mediators at the protein level (original data are provided in S2 Table) followed their changes in mRNA expression, except for TNF-α and CXCL3 in the ipsilateral hippocampus in which the concentrations of these proteins did not differ from those found in the contralateral tissue (Figs 3 and 4). An increase in brain synthesis of neutrophil chemoattractant CXCL1 observed after mTBI was associated with an elevation in its concentration in peripheral blood plasma at 6 h post-mTBI when compared to sham-injured rats (Fig 5). Other chemokines investigated (CXCL2, CXCL3, and CCL2) could not be detected in peripheral blood plasma with the immunoassays used in this study. When serum from samples of peripheral blood was analyzed, high levels of CXCL1 (~10-fold higher than those observed in plasma) were found in sham-injured rats, which did not change in response to injury (Fig 5).

### The influx of inflammatory cells into the injured brain after mTBI

**Fig 6:**
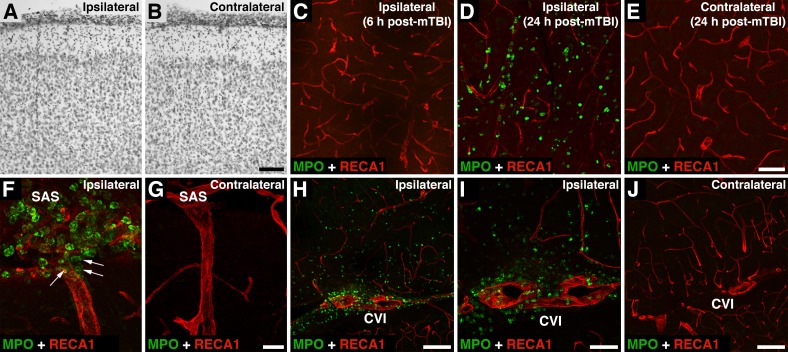
The influx of neutrophils into the injured brain after mTBI. In these experiments, the depth of brain deformation was set at 1 mm. Double immunostaining of injured brains was performed with anti-myeloperoxidase (MPO) antibody, a neutrophil marker, and an antibody to RECA-1, a marker for rat endothelial cells. (A, B) Histological analysis (cresyl violet staining) of the cerebral cortex at 4 weeks after mTBI. Note no apparent tissue damage with this severity of injury. (C–E) Unlike severe TBI, mTBI was not accompanied by any significant influx of neutrophils into the injured cortex until 24 h post-mTBI. (F, G) In contrast to brain parenchyma, a large number of neutrophils crossed the pial microvessels and invaded the subarachnoid space (SAS) near the injury site at 6 h post-mTBI. Note that some neutrophils entering the SAS appeared to subsequently move along the perivascular space to enter the brain parenchyma (arrows). (H–J) A robust influx of neutrophils into the ipsilateral cistern of velum interpositum (CVI), a slit-shaped cerebrospinal fluid space located above the 3rd ventricle with highly vascularized pia mater, at 6 h post-mTBI. Bars: panels A, B, H, J, 100 μm; panels C–E, I, 50 μm; panels F, G, 20 μm.

**Fig 7:**
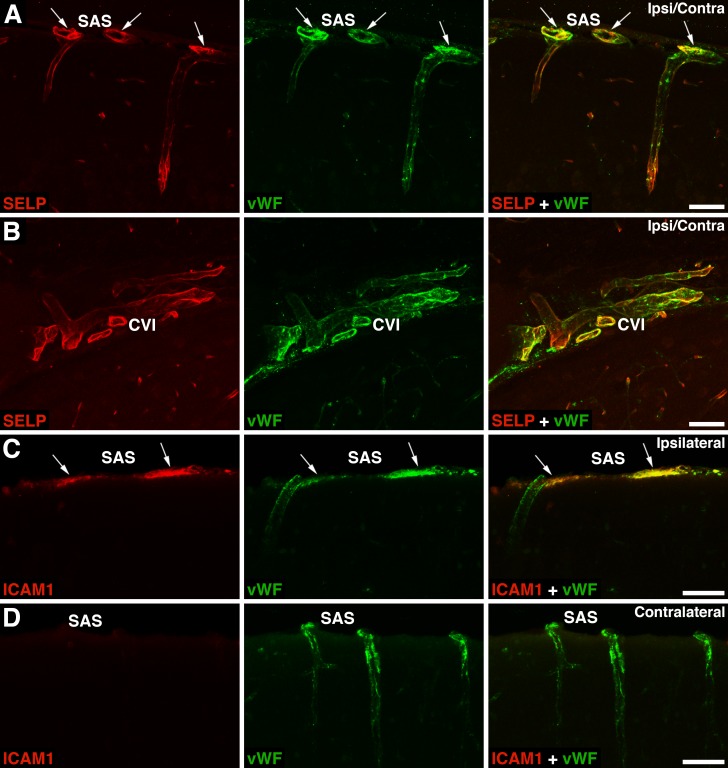
Expression of cell adhesion molecules in the injured brain after mTBI. In these experiments, the depth of brain deformation was set at 1 mm, and the images shown were acquired at 6 h post-mTBI. (A, B) Double immunostaining of injured brains with anti-P-selectin (SELP) antibody and an antibody to von Willebrand factor (vWF), a marker for endothelial cells. Note that SELP is expressed both ipsilaterally and contralaterally (Ipsi/Contra) on the endothelium of pial (arrows) and intraparenchymal microvessels. SELP was not expressed in the intact rat brain (data not shown). SAS and CVI are the subarachnoid space and the cistern of velum interpositum, respectively. (C, D) Double immunostaining of injured brains with anti-intercellular adhesion molecule 1 (ICAM1) antibody and an antibody to vWF. Note that ICAM1 is expressed on the endothelium of the ipsilateral pial (arrows), but not intraparenchymal, microvessels. ICAM1 is also not expressed on the endothelium of the contralateral pial microvessels. Bars: panels A–D, 50 μm.

The immunohistochemical analysis of injured brain tissue was performed in rats in which the depth of brain deformation was set at 1 mm. The histological analysis of the cerebral cortex at 4 weeks after mTBI showed no apparent signs of tissue damage with this severity of injury (compare Fig 6A and 6B). Unlike severe TBI in which a large number of neutrophils invade brain parenchyma as early as 6–8 h after injury [18,19], mTBI was not accompanied by any significant influx of these inflammatory cells into the injured cortex until 24 h post-mTBI (Fig 6C–6E). In contrast to brain parenchyma, a large number of neutrophils were found to cross the pial microvessels and invade the subarachnoid space (SAS) near the injury site at 6 h post-mTBI (Fig 6F and 6G). This correlated with an induction of expression of ICAM1 on the endothelium of the ipsilateral pial, but not intraparenchymal, microvessels at 6 h after injury (Fig 7C and 7D). Interestingly, at the same time point post-mTBI, another cell adhesion molecule SELP was expressed both ipsilaterally and contralaterally on the endothelium of pial and intraparenchymal microvessels (Fig 7A and 7B). SELP was not expressed in the intact rat brain (data not shown). At 6 h post-mTBI, we also observed a robust influx of neutrophils into the ipsilateral cistern of velum interpositum (CVI), a slit-shaped cerebrospinal fluid (CSF) space located above the 3rd ventricle with highly vascularized pia mater (Fig 6H–6J). From SAS and CVI, some neutrophils appeared to move along the perivascular spaces to enter the brain parenchyma (Fig 6F).

**Fig 8:**
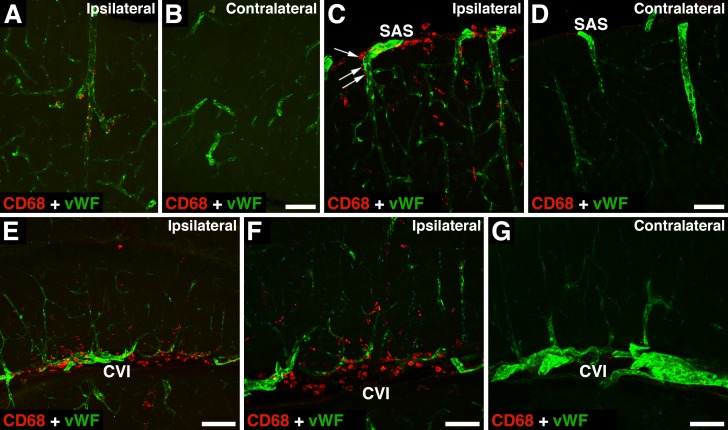
The influx of monocytes into the injured brain after mTBI. In these experiments, the depth of brain deformation was set at 1 mm, and the images shown were acquired at 24 h post-mTBI. Double immunostaining of injured brains was performed with anti-CD68 antibody, a monocyte marker, and an antibody to von Willebrand factor (vWF), a marker for endothelial cells. (A, B) Similar to severe TBI, in mTBI, monocytes did not appear in the injured brain until 24 h after the impact. However, only a small number of monocytes invaded the injured brain parenchyma at 24 h post-mTBI. Note that at this time point after injury, these inflammatory cells were predominantly located close to intraparenchymal blood microvessels. (C, D) In contrast to brain parenchyma, a large number of monocytes crossed the pial microvessels and invaded the subarachnoid space (SAS) near the injury site. Note that some of these monocytes appeared to subsequently move along the perivascular space to enter the brain parenchyma (arrows). (E–G) An influx of monocytes into the ipsilateral cistern of velum interpositum (CVI) also occurred at 24 h post-mTBI. Bars: panels A, B, E, 100 μm; panels C, D, F, G, 50 μm.

Similar to severe TBI [17], in mTBI, monocytes did not appear in the injured brain until 24 h after the impact. However, at 24 h post-mTBI, only a small number of monocytes were found to invade the injured brain parenchyma and they were predominantly located close to intraparenchymal blood microvessels (Fig 8A and 8B). At the same time, a large number of monocytes crossed pial microvessels, invading the SAS near the injury site and the ipsilateral CVI (Fig 8C–8G). Similar to neutrophils, some monocytes appeared to leave these CSF compartments, moving along the perivascular spaces to enter the brain parenchyma (Fig 8C).

**Fig 9:**
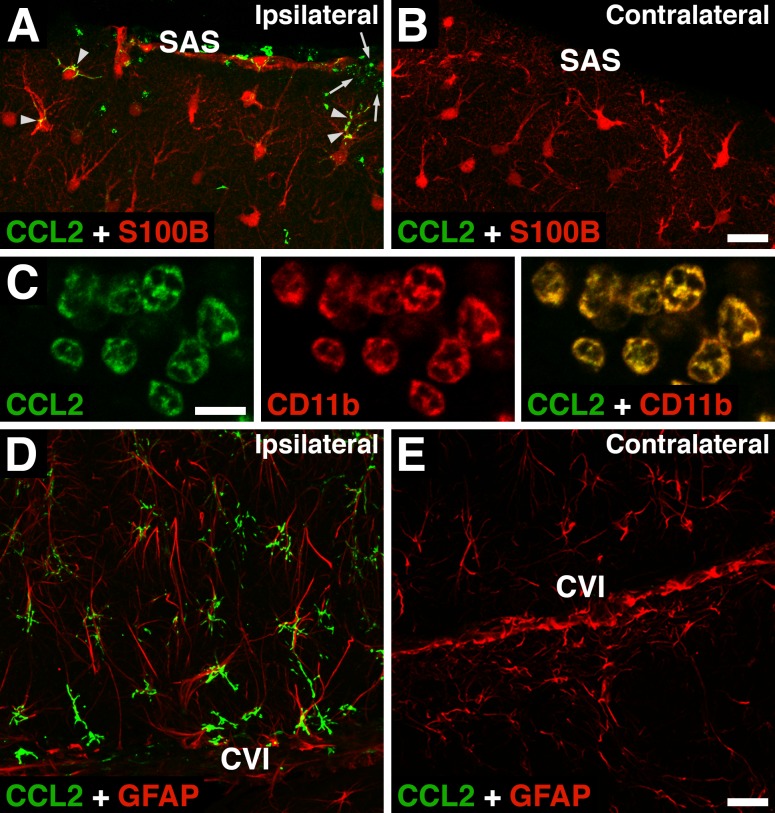
Expression of monocyte chemoattractant CCL2 in the injured brain after mTBI. In these experiments, the depth of brain deformation was set at 1 mm, and the images shown were acquired at 6 h post-mTBI. (A, B) Double immunostaining of injured brains with anti-CCL2 antibody and an antibody to S100B, an astrocyte marker. Note that CCL2 is not only expressed ipsilaterally in astrocyte processes (arrowheads), but is also carried by neutrophils initially entering the subarachnoid space (SAS) near the injury site and then invading the injured brain parenchyma (arrows). (C) Double immunostaining of neutrophils entering the SAS with anti-CCL2 antibody and an antibody to CD11b, a neutrophil marker. (D, E) The astrocytic expression of CCL2 in the vicinity of the cistern of velum interpositum (CVI). Double immunostaining of injured brains was performed with anti-CCL2 antibody and an antibody to glial fibrillary acidic protein (GFAP), an astrocyte marker. Bars: panels A, B, D, E, 25 μm; panel C, 10 μm.

In our previous study involving the rat model of severe TBI [17], we have shown that at the early stage post-injury (6 h after TBI), the cerebrovascular endothelium in the injured brain parenchyma produces neutrophil and monocyte chemoattractants. In mTBI, however, we were not able to detect these chemokines in the cerebrovascular endothelium in the injured brain parenchyma using immunohistochemistry (data not shown). Interestingly, while astrocytes were not a significant source of CXC chemokines in severe TBI [17], they highly expressed CCL2 after both severe and mild TBI (Fig 9A, 9B, 9D and 9E). CCL2 was also highly expressed in neutrophils invading the injured brain at 6 h after mTBI (Fig 9A–9C), but not at 24 h post-mTBI (data not shown).

## Discussion

In the present study, a rat model of mTBI was employed that was similar to the model of focal mTBI previously used by Redell et al. [10]. Brain injury sustained in this model resembles complicated mTBI/concussion in humans [20]. However, it is important to note that a rapid and substantial increase in brain production of proinflammatory mediators reported in this paper is also typical of a more commonly used model of diffuse mTBI [9,10]. In the present study, we observed an early (within 6 h post-mTBI) increase in synthesis of proinflammatory cytokines, chemokines, and MMP9 in the injured cortex and hippocampus that was limited to the first 24 h after the impact. Interestingly, the synthesis of CHI3L1, a proinflammatory mediator playing a pathophysiological role not only in inflammation, but also in carcinogenesis and tissue injury and remodeling [21], increased only slightly at 6 h post-mTBI, but quite substantially at 24 h after injury. Using the CCI model of severe TBI, Bonneh-Barkay at al. [22] have demonstrated a prolonged (beyond first week post-TBI) upregulation of message for CHI3L1 and identified astrocytes as the major source of this protein in the injured brain. These investigators also showed that in patients with severe TBI, the increased levels of CHI3L1 in the CSF are maintained for at least one week after injury. Given its multiple biological actions, CHI3L1 may play a role either in augmenting secondary injury or in post-injury repair processes by, for example, inducing alternative M2 macrophage differentiation [21]. An upregulation of brain synthesis of MMP9 within the first 6 h post-mTBI was consistent with our previous clinical study demonstrating an increase in plasma MMP9 levels in patients sustaining a concussion [8]. Rodent studies of severe TBI [23,24] suggest that MMP9 may also represent a potential target for therapeutic intervention in mTBI/concussion.

In addition to assessing changes in cortical and hippocampal synthesis of chemokines after mTBI, we investigated whether these changes would also be reflected in peripheral blood. Knowing that some chemokines are carried by circulating leukocytes and/or are bound to Duffy antigen receptors expressed on erythrocytes [25,26], we asked whether there would be differences in their serum versus plasma levels due to the possible release of these proinflammatory mediators during the coagulation process. Serum concentration of CXCL1 in sham-injured rats was 10 times higher than that in plasma, suggesting that this chemokine could have been released from its cellular sources during clot formation. CXCL1 is an acute phase protein and its liver synthesis was shown to be upregulated after TBI [27]. Thus, it is possible that an elevation of plasma concentration of CXCL1 found after mTBI was also associated with increased production of this chemokine in the liver. Our observations suggest that the choice between analyzing serum versus plasma levels of certain proinflammatory mediators is not trivial and could affect the results obtained and their interpretation.

Consistent with increased production of chemokines after mTBI, we observed an influx of neutrophils and monocytes into the injured brain. A new finding of this study is that at the early stage post-mTBI, leukocytes predominantly invaded the injured brain across pial microvessels, initially entering the CSF-filled SAS and CVI. While pial vessels are continuous with intraparenchymal blood vessels, their anatomical location within the SAS [28] and, presumably, CVI suggests that they are a part of the blood-CSF rather than the blood-brain barrier (BBB). Several lines of evidence indicate that there are functional differences between the pial and intraparenchymal microvessels. Clearly, pial microvessels lack the perivascular astrocytic ensheathment that is typical of brain intraparenchymal microvessels [29], and astrocytes have the ability to functionally affect the cerebrovascular endothelium via paracrine signaling [30,31]. The endothelium of pial microvessels can produce neutrophil and monocyte chemoattractants [32], and peripheral administration of TNF-α or lipopolysaccharide in mice has been shown to induce a strong expression of SELP on the endothelial surface of pial microvessels, but a much weaker expression on endothelium of intraparenchymal microvessels [33]. It has also been demonstrated that the intraparenchymal injection of IL-1β causes acute recruitment of inflammatory cells to meninges, but does not result in leukocyte invasion at the site of injection [34]. In mice intracranially infected with the parasite *Mesocestoides corti*, the predominant influx of inflammatory cells across the pial microvessels was also observed, which occurred within a much shorter time frame after infection than the leukocyte recruitment across the intraparenchymal microvessels [35]. Similarly, in experimental autoimmune encephalomyelitis (EAE), an animal model of multiple sclerosis, the leukocyte invasion of meninges precedes the leukocyte influx into the brain parenchyma [36].

The initial influx of neutrophils into the ipsilateral SAS and CVI coincided with an early induction of expression of ICAM1 on the endothelium of the ipsilateral pial, but not intraparenchymal, microvessels. While the ICAM1-positive immunostaining was observed only in the injured side of the brain, SELP, a cell adhesion molecule involved in the capture and rolling of leukocytes [37], was expressed both ipsilaterally and contralaterally on the endothelium of pial and intraparenchymal microvessels. SELP was not expressed in the intact brain, suggesting that unlike in humans, in rats, this cell adhesion molecule is not stored in the Weibel-Palade bodies and its endothelial expression is induced in response to injury [38]. This bilateral induction of SELP expression observed in mTBI suggests that chemokines act as the primary guidance cues for inflammatory cells to invade the injured brain. This idea is consistent with observations that the post-injury synthesis of neutrophil and monocyte chemoattractants was upregulated only in the ipsilateral cortex and hippocampus.

Our observations suggest that neutrophils and monocytes initially entering the CSF-filled SAS and CVI may then migrate into the brain parenchyma along the perivascular (Virchow-Robin) spaces (see Figs 6F and 8C). Similar movement of inflammatory cells along the perivascular spaces from the CSF compartment into the brain parenchyma has been demonstrated in EAE [39]. Such movement of leukocytes would require the presence of the chemotactic gradient, and may be, at least in part, dependent on junctional adhesion molecule JAM-A, a component of the tight junction complexes at the BBB, which is also expressed on the surface of inflammatory cells [40].

The neutrophil influx into the SAS and CVI observed at 6 h post-mTBI coincided with an early increase in cortical and hippocampal expression of neutrophil chemoattractants CXCL1–3. However, the synthesis of these chemokines in the injured brain declined to the pre-injury levels within 24 h after mTBI, a time point at which a large number of neutrophils invaded the brain parenchyma. Similarly, CCL2, a major monocyte chemoattractant, was highly expressed in astrocytes and neutrophils at 6, but not 24 h, post-mTBI, whereas monocytes invaded the SAS and CVI not earlier than one day after injury. This apparent lack of correlation between the time course of changes in production of neutrophil and monocyte chemoattractants and the timing of influx of inflammatory cells suggests that other than investigated here chemokines played a role in leukocyte invasion observed after mTBI. The nature of these putative chemokines is currently unclear; however, it is possible that CHI3L1 whose brain synthesis is upregulated for a prolonged period of time after neurotrauma can act as a monocyte chemoattractant in the injured brain [41].

In summary, we have shown that mTBI is associated with a rapid and robust brain inflammatory response to injury. At the early stage post-mTBI, the invading inflammatory cells predominantly enter the CSF-filled compartments crossing the pial rather than intraparenchymal microvessels, from where they appear to migrate into the brain parenchyma along the perivascular spaces. These observations corroborate the hypothesis that the pial vessels differ functionally from intraparenchymal vasculature. Our findings also suggest that neuroinflammation associated with mTBI could potentially be targeted therapeutically especially in individuals who are likely to exhibit delayed recovery from injury [4–7]. While the objective diagnostic tests to identify these individuals are yet to be established, the idea of targeting neuroinflammation in such patients is supported by the data from animal studies of severe TBI in which the pharmacologic (administration of anti-CD11d or anti-Ly6g antibodies) or genetic (deletion of the *Cxcr2* or *Ccl2* gene) manipulations aimed at reducing the influx of neutrophils and/or monocytes have been shown to decrease the post-traumatic loss of neural tissue and improve functional outcome after injury [11–15].

## Supporting Information

S1 Table Original RT-PCR data. (XLSX)

S2 Table Original ELISA data. (XLSX)
